# Transcriptional profiling of the Arabidopsis abscission mutant *hae hsl2* by RNA-Seq

**DOI:** 10.1186/1471-2164-14-37

**Published:** 2013-01-17

**Authors:** Chad E Niederhuth, O Rahul Patharkar, John C Walker

**Affiliations:** 1 Division of Biological Sciences, University of Missouri, Columbia, Missouri 65211, USA; 2 Bond Life Sciences Center, University of Missouri, Columbia, Missouri 65211, USA; 3 Interdisciplinary Plant Group, University of Missouri, Columbia, Missouri 65211, USA

## Abstract

**Background:**

Abscission is a mechanism by which plants shed entire organs in response to both developmental and environmental signals. *Arabidopsis thaliana*, in which only the floral organs abscise, has been used extensively to study the genetic, molecular and cellular processes controlling abscission. Abscission in Arabidopsis requires two genes that encode functionally redundant receptor-like protein kinases, *HAESA* (*HAE*) and *HAESA-LIKE 2* (*HSL2*). Double *hae hsl2* mutant plants fail to abscise their floral organs at any stage of floral development and maturation.

**Results:**

Using RNA-Seq, we compare the transcriptomes of wild-type and *hae hsl2* stage 15 flowers, using the floral receptacle which is enriched for abscission zone cells. 2034 genes were differentially expressed with a False Discovery Rate adjusted p < 0.05, of which 349 had two fold or greater change in expression. Differentially expressed genes were enriched for hydrolytic, cell wall modifying, and defense related genes. Testing several of the differentially expressed genes in *INFLORESCENCE DEFICIENT IN ABSCISSION* (*ida*) mutants shows that many of the same genes are co-regulated by IDA and HAE HSL2 and support the role of IDA in the HAE and HSL2 signaling pathway. Comparison to microarray data from stamen abscission zones show distinct patterns of expression of genes that are dependent on *HAE HSL2* and reveal *HAE HSL2*- independent pathways.

**Conclusion:**

*HAE HSL2*-dependent and *HAE HSL2*-independent changes in genes expression are required for abscission. *HAE* and *HSL2* affect the expression of cell wall modifying and defense related genes necessary for abscission. The *HAE HSL2*-independent genes also appear to have roles in abscission and additionally are involved in processes such as hormonal signaling, senescence and callose deposition.

## Background

Abscission is the programmed separation of plant organs [1-5]. It is both a developmental and environmental response, allowing non-functioning or infected organs to be discarded. Abscission occurs at a specialized cell layer called the Abscission Zone (AZ), which develops at the base of the abscising organ. Secretion of hydrolytic and cell wall modifying enzymes in the AZ results in the breakdown of the pectin-rich middle lamella, leading to organ separation. This is followed by the formation of a protective scar-layer of waxy substances over the newly exposed AZ.

In *Arabidopsis thaliana* the floral organs abscise soon after pollination. Abscission requires the function of two receptor-like protein kinases (RLK) encoded by the genes *HAESA* (*HAE*) and *HAESA-LIKE 2* (*HSL2*). While single mutants appear phenotypically normal, the double mutant has a complete loss of abscission [6-8]. Plants with mutations in *INFLORESCENCE-DEFICIENT IN ABSCISSION* (*IDA*) have similar phenotypes to *hae hsl2* mutants [6,9]. *IDA* is predicted to encode a small secreted protein [9] and has been placed upstream of *HAE* and *HSL2*, which suggests IDA is the putative ligand for HAE and HSL2 [6,7]. Plants expressing a double RNAi transgene targeting the MAP Kinase Kinases MKK4 and MKK5 also are defective in abscission and constitutively active versions of either *MKK4* or *MKK5* can rescue *hae hsl2* and *ida* mutants. The MAP Kinases MPK3 and MPK6 are known targets of MKK4/MKK5 and expression of dominant negative versions of MPK6 in a *mpk3* mutant display abscission defective phenotypes [6]. This evidence suggests a pathway consisting IDA, HAE, HSL2, and a MAP kinase cascade regulate the initiation of abscission.

The roles of polygalacturonases (PG), xylogulcan endo-transglycosylase/hydrolases (XTHs), and cellulases have been associated with abscission in tomatoes, cotton, and roses [10-13]. A previous microarray study of stamen abscission zones in Arabidopsis has shown that expression of many genes encoding hydrolytic and cell wall modifying enzymes are increased prior to abscission [14,15]. These include the PG encoding genes *PGAZAT/ADPG2 *[16,17] and *QRT2*[18]. Interestingly *adpg2/qrt2* double mutant plants have a delayed abscission phenotype [18] which provides functional evidence these PGs have a role in abscission.

Transcriptional profiling using high throughput next-generation sequencing (RNA-Seq) has emerged in recent years as a superior alternative to microarrays [19]. Here we report the use of RNA-Seq to identify differentially expressed genes in *hae hsl2* flower receptacles. Our work suggests that *HAE* and *HSL2* act to promote the expression of hydrolytic and cell wall modifying enzymes necessary for abscission and that disruption of the HAE HSL2 signaling pathway results in reduced expression of these enzymes explaining the loss of abscission phenotype in the *hae hsl2* mutants.

## Results and discussion

### RNA-Seq of wild type and *hae hsl2* receptacles

Arabidopsis flower development can be broken down into 20 developmental stages based on morphology [20]. Abscission studies typically focus on stages 12-17 (Additional file 1), with organ loss occurring at stage 17. At stage 15 the floral organs are still attached, but by stage 16 the organs have begun to wither and will fall if force is applied. By stage 17 all the floral organs have abscised from the still green siliques. Expression of *HAE, HSL2*, and *IDA* in floral AZs increases from stage 12 reaching its peak in the latter parts of stage 15 [14]. Based on these observations we hypothesized that initiation of abscission occurs during stage 15 and that expression differences between wild type and *hae hsl2* would be observed at this point.

AZs constitute only a small part of the flower. Flower receptacles, defined as the region of the flower stalk where the floral organs are attached, were dissected from stage 15 flowers to enrich for AZ RNA (Additional file 1). RNA-seq libraries were constructed for three biological replicates of wild type and *hae hsl2* flower receptacles. These six samples were then multiplexed and sequenced on three lanes of an Illumina HiSeq 2000. Multiplexing barcoded samples and sequencing across multiple lanes means that lane to lane technical variation will apply to all samples with no loss of sequencing depth [21]. A total of 270,077,377 reads passed a quality filter and were mapped back to the Arabidopsis TAIR10 genome (Table 1). Approximately 97% of all reads were mapped back, of which 87% mapped uniquely to only one location and could be assigned to a single annotated TAIR10 gene.

**Table 1:** RNA-Seq reads and mapping statistics

**Sample**	**Total Reads**	**Reads Mapped**	**Percent Mapped**	**Uniquely Mapped to a Single Gene**	**Percent Uniquely Mapped to a Single Gene**
WT 1	45,281,039	44,356,413	97.958	39,461,618	87.148
WT 2	56,387,916	55,580,136	98.567	48,931,246	86.776
WT 3	44,946,010	43,804,264	97.460	39,400,029	87.661
*hae hsl2* 1	42,997,215	42,107,956	97.932	37,448,466	88.934
*hae hsl2* 2	34,165,472	33,425,452	97.834	29,705,403	86.946
*hae hsl2* 3	46,299,725	45,529,680	98.337	40,673,161	87.848
Total	270,077,377	264,803,901	98.047	235,619,923	87.242

**Figure 1:**
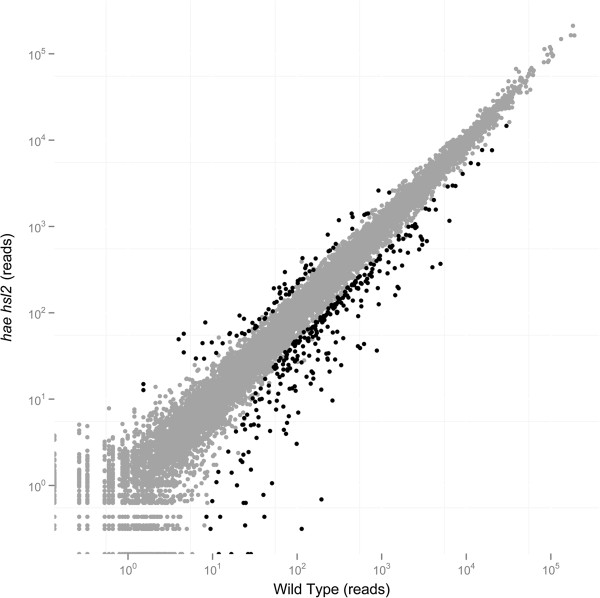
**Scatter plot of mean normalized read counts for wild type versus *hae hsl2*.** Black data points indicate genes that show 2 fold or greater changes in expression with a FDR adjusted p < 0.05, R^2^ = 0.987 (Pearson’s Correlation).

Read counts were normalized and differential gene expression was examined for the uniquely mapped reads using the DESeq package [22] (Additional file 2A). A global comparison of the samples shows a very high correlation (R^2^ = 0.987, Pearson’s Correlation Coefficient) between wild type and *hae hsl2* (Figure 1). Genes were determined to be differentially expressed with a False Discovery Rate (FDR) adjusted p-value < 0.05 giving 2034 differentially expressed genes. 1221 of the differentially expressed genes having lower expression in the receptacles of *hae hsl2* plants and 813 having higher expression in the double mutant. For this study we chose to focus on those differentially expressed genes that showed 2 fold or greater changes in expression (Additional file 2B). This resulted in the selection of 349 differentially expressed genes, 277 of which were lower in *hae hsl2* and 72 were higher in mutant plants (Figure 1).

The global transcriptome changes observed when comparing wild type to *hae hsl2* floral receptacles suggest several conclusions. First, the high correlation between all samples shows that there was little variation in the collection of floral receptacles and that these were technically consistent. Secondly, expression of the majority of genes is unaffected in *hae hsl2*, suggesting that this pathway targets a limited number of genes. This is supported morphologically as stage 15 *hae hsl2* flowers appear normal and have normal looking AZs [6]. Finally, the majority of the differentially expressed genes are lower in the mutant relative to wild type. This suggests the HAE HSL2 signaling pathway primarily activates expression of target genes.

### Molecular and biological functions of differentially expressed genes

A global view of the functions of the differentially expressed genes and the underlying biology can be obtained by examining their gene ontology (GO) [23]. This is a system that categorizes genes into groups of terms based on their predicted or experimentally derived Molecular Function, Biological Process, and Cellular Component. Molecular Function includes the most genes and is usually based on sequence similarity. The Biological Process categories are typically derived empirically and as a result tend to be more stringent, but also limited, having fewer annotated genes. The Cellular Component is largely a prediction of where the gene product is localized. Looking for overrepresentation of GO terms indicates that more genes in a list are represented in that category than what would be expected by random chance and can reveal trends in the data.

**Figure 2:**
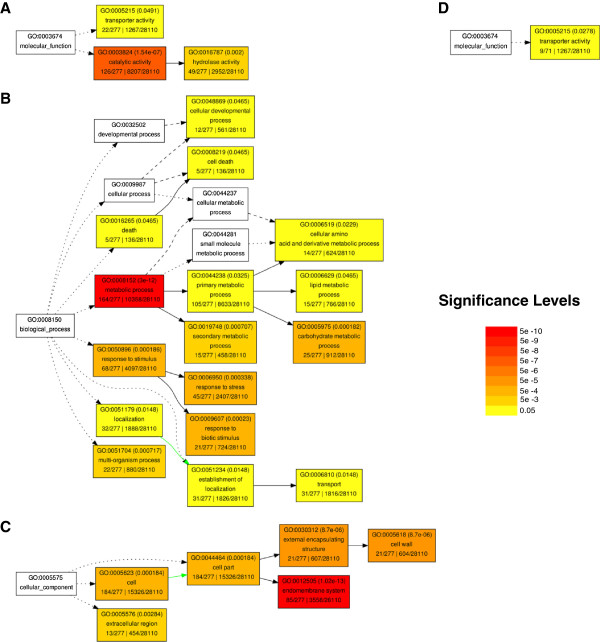
**GO Slim Plant term enrichment.** Genes with lower expression in *hae hsl2 ***(A)** Biological Process **(B)** Molecular Function **(C)** Cellular Component. GO Slim Plant term enrichment of genes with higher expression in hae hsl2 **(D)** Molecular Function. Color Scale represents FDR adjusted p-values < 0.05. Solid, dashed, and dotted lines represent either two, one, or zero enriched terms at either end of the line.

In the genes that are expressed at lower levels in the *hae hsl2* mutants, the biological process category has enrichment for carbohydrate metabolic process (Figure 2A) and cell wall modification (Additional file 3A). There is also enrichment for terms related to stress responses, particularly to biotic stimulus and secondary metabolic processes, as well as secondary metabolism such as phenylpropanoid metabolism. The molecular functions of genes showing lower expression in the mutant were enriched for those having hydrolytic activity (Figure 2B). A majority of the hydrolases in this subset act on glycosyl bonds and include PGs, XTHs, glycosyl hydrolases, beta glucosidases, cellulases, and chitinases (Additional file 3A). The hydrolases also include pectinesterases, which work in conjunction with PGs to break down the glycosidic bonds of pectin, a main component of the middle lamella. Although not meeting the 5 gene minimum cutoff for GO term enrichment, pectate lyases are also found within the genes that have lower expression in *hae hsl2*.

Genes showing higher expression in the mutants relative to wild type were enriched for lipid metabolism and transporter activity (Figure 2D). The genes that are annotated to have a role in transporter activity are primarily involved with either ion transport or secondary active transmembrane transport (Additional file 3B).

The genes with lower expression in *hae hsl2* largely fall in one of two categories based on GO term enrichment; those involved with middle lamella/cell wall degradation and remodeling, and those involved in defense against pathogens. Many of the hydrolases identified as having lower expression in the *hae hsl2* samples are also predicted to be localized to the cell wall or endomembrane system (Figure 2C), and are annotated as being involved in cell wall modification (Additional file 3A). In particular, this group of genes encodes the enzymes that breakdown pectin, the PGs, pectinesterases, and pectin lyases, that are necessary for cell separation [1-5]. In the case of the PGs, two have been previously shown to be involved in abscission [16-18], *PGAZAT/ADPG2* and *QRT2*. These results show that HAE and HSL2 regulate the breakdown of the middle lamella during abscission. Shedding of an organ also exposes a fresh surface that is potentially susceptible to infection. It has been previously proposed [4] that plants activate defense responses as a protective measure prior to abscission. This idea is supported by our results where failure to abscise coincides with relatively lower expression of defense related genes that have roles in response to biotic stressors, particularly bacteria and fungi. After the organ has abscised protective layers of suberin and lignin form over the AZ [2]. Several genes involved in the biosynthesis of the phenylpropanoid precursors of these compounds have lower expression in *hae hsl2* mutants. For example, the fatty acyl-coenzyme A reductases *FAR4* and *FAR5* and the acyltransferase *GPAT5* are essential to the biosynthesis of suberin [24,25] and show lower expression in *hae hsl2*, along with three genes involved in lignin biosynthesis (Additional file 3A). The possible roles of the genes that are expressed at relatively higher levels in the *hae hsl2* mutants are less clear. Many of the genes are predicted to function in ion and secondary active transmembrane transport. Potentially these may be involved in the transport of nutrients to the floral organs and are switched off prior to or during abscission.

### Expression patterns in stamen AZ

Previous microarray studies have examined gene expression changes in stamen AZs across flower development stages 12, 13 and early, mid, and late stage 15 [14]. In their analysis Cai and Lashbrook [14] used a linear mixed model to identify 551 genes in eight expression clusters that showed the most significant changes across all developmental stages. Because this analysis focused on changes in gene expression over time many of the genes they identified as having differential expression are not differentially expressed in *hae hsl2* relative to wild type. We reanalyzed the raw microarray data to determine the wild type expression patterns of the differentially expressed genes identified from the RNA-seq experiments. The Arabidopsis ATH1 Genome Array used by Cai and Lashbrook with TAIR10 annotations has probes for 21,144 genes. Only 219 of the 277 genes with lower expression in the double mutant and 53 of the 72 genes with higher expression are found in the microarray expression data and all subsequent analysis was done using these smaller datasets.

**Figure 3:**
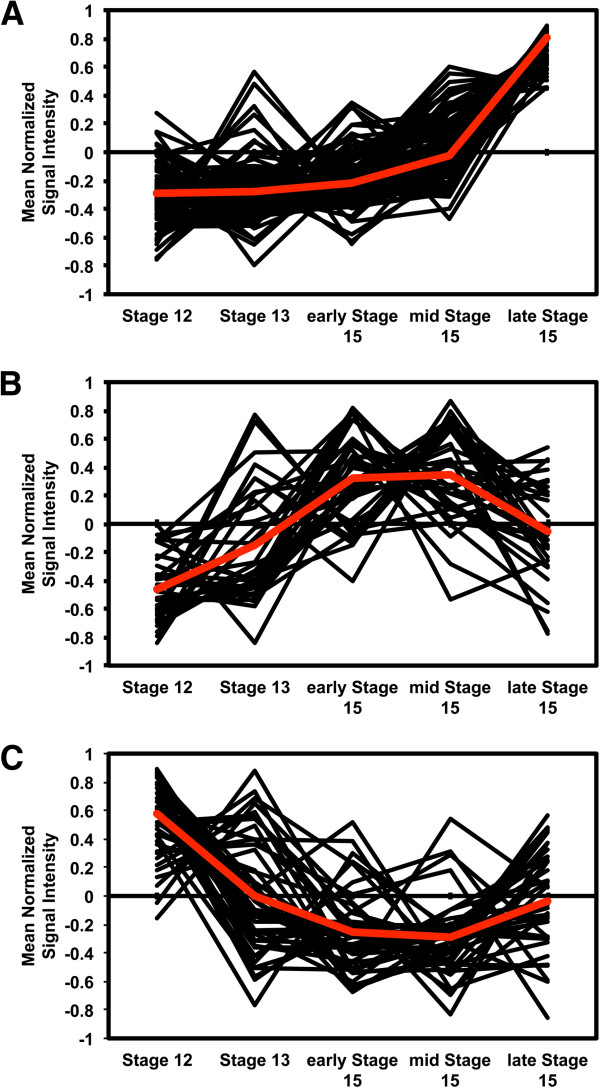
**Expression patterns of genes with lower expression in *hae hsl2 *in Stamen AZ microarrays. (A)** Cluster 1 **(B)** Cluster 2 **(C)** Cluster 3.

The 219 genes that are expressed at lower levels in the double mutant relative to wild type were clustered into three groups by the k-means algorithm (Figure 3 and Additional file 4), which was determined to be the most representative number of clusters using the gap statistic [26] (Additional file 5). The majority of genes, 144 out of 219, fall into cluster 1 (Figure 3A) which is characterized by lower expression in stages 12 to mid-stage 15 and increasing considerably in late stage 15. GO term-enrichment analysis shows that this cluster is enriched for terms that would be involved in cell wall modification (Additional file 6A). The biological process category contains genes for membrane transport, cell wall organization, and carbohydrate metabolic processes. There is also additional enrichment for defense responses such as response to bacterium and response to fungus. In the molecular function category nearly all the differentially expressed hydrolases, particularly the glycosyl hydrolases, pectinesterases, and genes with glycosyl transferase activity are in cluster 1. While the cellular component is enriched for localization to the endomembrane system, cell wall, and extracellular region.

Cluster 2 (Figure 3B), with 33 genes, shows relatively low expression in stages 12 and 13, increases through early and mid-stage 15 and decreases slightly in late stage 15. Transmembrane transport and oxidation reduction are found in the biological process category for cluster 2 (Additional file 6B). Similarly in the molecular function there is enrichment for electron carrier activity, oxidoreductase activity, and transporter activity, particularly ion transport. Many of these genes are localized to the endomembrane system or to the membrane and these terms show enrichment in this cluster for the cellular component.

Cluster 3 (Figure 3C) has 42 genes which show relatively higher expression in stage 12, reduced expression through early and mid-stage 15 and an increase in expression in late stage 15. Although Cluster 3 has peak expression in stage 12, the expression profiles across stages 13 to late stage 15 is similar to Cluster 1. This cluster shows the least overrepresentation of GO terms (Additional file 6C). In biological process there is enrichment for oxidation reduction, carbohydrate metabolic process, and cellular amino acid and derivative metabolic process. There was no enrichment in either cellular component or molecular function.

The dynamics of expression in these clusters and the functions of their genes show that most genes affected by HAE HSL2 have relatively lower expression in earlier flower development stages when *HAE*, *HSL2*, and *IDA* expression is also low. In later developmental stages, the first genes to that display an increase in expression tend to be those involved in transmembrane transport and oxidative reduction. It is not until late in stage 15 that there is activation of the genes associated with cell separation, defense responses, and the formation of the protective scar tissue over the newly exposed AZ.

## QPCR results

**Figure 4:**
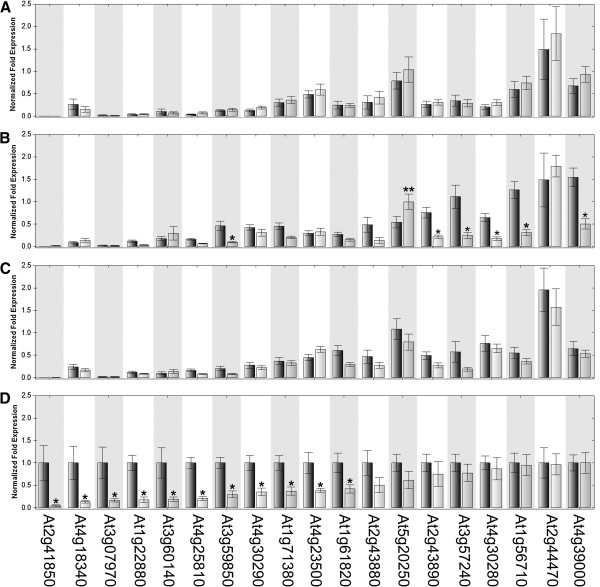
**qPCR of hydrolases with lower expression in *hae hsl2 *(grey bars) RNA-Seq data in comparison to wild type (black bars). (A)** stage 12 flower receptacles **(B)** stage 13 flower receptacles **(C)** stage 14 flower receptacles **(D)** stage 15 flower receptacles. (*) significantly lower in *hae hsl2* (**) significantly higher in *hae hsl2*. Error bars are standard error of the mean.

We used qPCR to confirm the expression patterns and differences of 19 glycosyl hydrolases identified as having lower expression in *hae hsl2* (Figure 4). These were done on flower receptacle RNA isolated from an independent set of biological replicates and done across stages 12, 13, 14, and 15 (Additional file 1). These results show that 11 of the 19 hydrolases have lower expression with a p-value < 0.05 between wild type and *hae hsl2* at stage 15. Interestingly several of those that are not statistically significant at stage 15 are in stage 13 *hae hsl2* receptacles, suggesting *hae hsl2* may have affects on gene expression at earlier stages. Looking at the patterns of expression across the 4 stages, most of the hydrolases have significantly lower expression prior to stage 15 (Additional file 7A), which is consistent with the expression patterns observed in stamen AZs [14]. The patterns of expression observed by qPCR in flower receptacles are similar to those in stamen AZ microarrays, showing that gene expression changes in the AZ are observable in receptacles.

**Figure 5:**
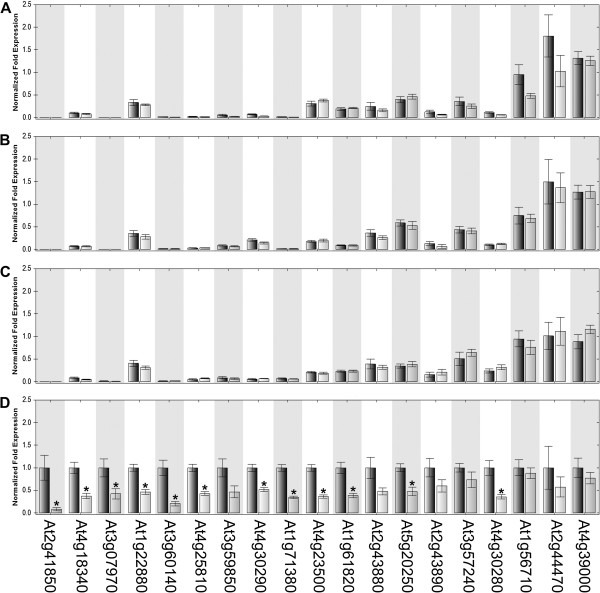
**qPCR of hydrolases with lower expression in *ida *(grey bars) RNA-Seq data in comparison to wild type (black bars). (A)** stage 12 flower receptacles **(B)** stage 13 flower receptacles **(C)** stage 14 flower receptacles **(D)** stage 15 flower receptacles. (*) significantly lower in *ida* (**) significantly higher in *ida*. Error bars are standard error of the mean.

IDA is a putative ligand of HAE and HSL2 and it would be predicted that *ida* mutants affect the same target genes as *hae hsl2*. We tested this hypothesis by doing qPCR for the 19 glycosyl hydrolases across the four developmental time points (Figure 5) on *ida* mutant receptacles and a new set of wild type receptacles. Across stages 12-15, the results are replicable in wild type (Additional file 7A and C) while *ida* and *hae hsl2* have very similar expression profiles (Additional file 7B and D). At stage 15, 12 glycosyl hydrolases were confirmed as differentially expressed in *ida* mutants relative to wild type, including the 10 of the 11 confirmed by qPCR in *hae hsl2*. The differences between *ida* and *hae hsl2* are likely due to technical variation between qPCR experiments. This shows that *ida* and *hae hsl2* affect the expression of many of the same genes and co-regulate these genes during abscission. This further supports the proposed pathway where IDA is the putative ligand of HAE and HSL2. That these same genes are differentially expressed in two different abscission mutants serves as further evidence of their importance in abscission.

### Identification of *HAE HSL2* -independent genes

**Figure 6:**
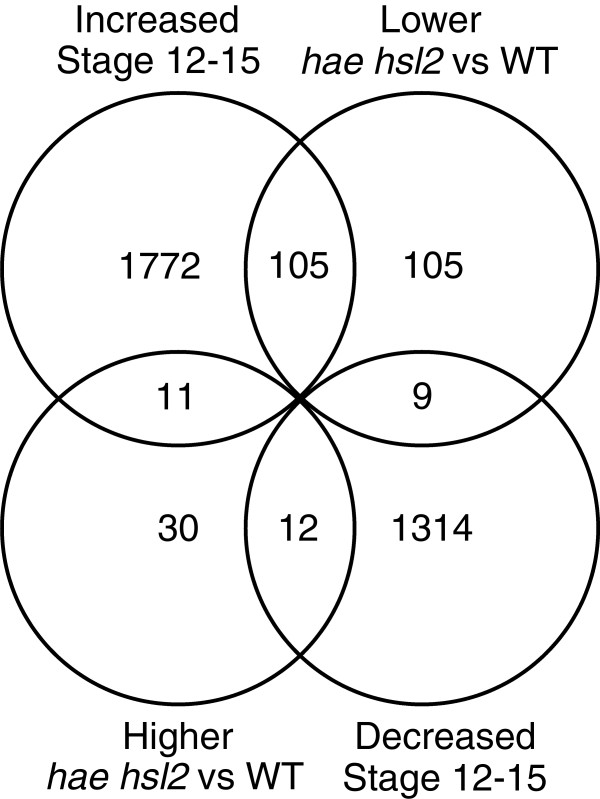
Overlap of RNA-Seq differentially expressed genes with stamen AZ stage 12-late stage 15 differentially expressed genes.

The RNA-Seq results identify genes affected by *hae hsl2* which can be classified as *HAE HSL2*-dependent genes. Again using the stamen AZ microarray data with the RNA-Seq results it is possible to identify *HAE HSL2*-independent genes with potential roles in abscission. Stamen AZ microarrays were reanalyzed for differential expression across developmental stages using stage 12 as the baseline for expression. These lists of differentially expressed genes were then compared to the lists of RNA-Seq differentially expressed genes for overlap, only those genes from the RNA-Seq with probes on the array were used for comparison (Additional file 8A and B). The z-score indicates the statistical significance of the overlap between gene lists (Additional file 8C) which can be determined given the background list of all Arabidopsis genes. The most significant overlap was between the lower expressed *hae hsl2* genes and those increasing in expression from stage12-late stage 15 (Figure 6), which corroborates the k-means cluster results and further supports the conclusion that the HAE HSL2 signaling pathway primarily activates gene expression.

**Figure 7:**
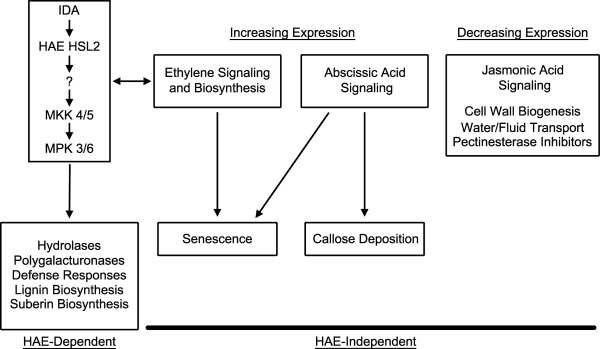
**HAE HSL2 signaling pathway and a summary of *HAE HSL2*-dependent and *HAE HSL2*-independent gene expression changes during abscission.**

To identify *HAE HSL2*-independent genes we focused on the genes differentially expressed between stage 12 and late stage 15. The list of candidate *HAE HSL2*-independent genes includes 1772 genes that increase in expression between stage 12 and late stage 15 stamen AZs and 1314 genes whose expression decreases. The functions of these genes were then examined to understand *HAE HSL2*-independent processes (Figure 7).

Genes involved in several hormonal signaling pathways show increased expression at late stage 15 (Additional file 9A). Of particular interest are those involved in ethylene biosynthesis and signaling, as ethylene regulates the timing of floral organ abscission [1,3]. This includes several ethylene biosynthesis genes such as the s-adenosylmethionine synthetase *SAM1*, the ACC synthase *ACS8*, and two ACC oxidases *ACO1* and *ACO2*. The ethylene receptor *ETR2* also increases in expression and is known to be inducible by ethylene signaling [27]. Expression of genes associated with abscisic acid signaling also increase, such as the genes encoding protein phosphatase 2C *ABA INSENSITIVE 2* (*ABI2*) and the transcription factors *ABI4* and *ABI5*.

Both ethylene and abscisic acid regulate floral senescence [28] and markers of senescence show enrichment in the *HAE HSL2*-independent gene set. This corresponds to the senescence and withering of the floral organs during abscission. Callose deposition and localization also shows enrichment and is another response of abscisic acid signaling [29]. Physiologically this is supported by work that has shown increases in callose deposition in abscising leaves of *Phaseolus vulgaris *[30] and may be necessary to prevent water loss after abscission.

Jasmonic acid signaling and biosynthesis genes decrease in expression (Additional file 9B) from stage 12 to 15. This includes the allene oxide synthase *AOS*, the allene oxide cyclases *AOC3* and *AOC4*, and the lipoxygenases *LOX2* and *LOX4*. Interestingly *aos* mutants have a delayed abscission phenotype that is enhanced synergistically by mutations in the ethylene insensitive *ein2* and the ABA-deficient mutant *aba2 *[17]. Also decreasing in expression are cellulose biosynthesis genes such as *CELLULOSE SYNTHASE 5* (*CESA5*), *CELLULOSE SYNTHASE CATALYTIC SUBUNIT 7* (*CESA7*), and multiple cellulose synthase-like genes. This suggests there is inhibition of cell wall biogenesis while at the same time that cell wall modification is occurring. While pectinesterases increase in expression in a *HAE HSL2*-dependent manner during stage 15, pectinesterase inhibitor genes decrease in expression independently of *HAE HSL2*.

There is likely cross talk between *HAE HSL2*-dependent and *HAE HSL2*-independent processes, particularly by ethylene (Figure 6). Ethylene signaling may affect the timing and degree of *IDA* expression [31]. Additionally ethylene-dependent abscission signaling could be modulated by MAP kinase signaling [32] either through regulation of the ACC synthases and ethylene biosynthesis [33] or through the transcription factor EIN3 [34]. Some classes of genes are found to varying extents in both the *HAE HSL2*-dependent and *HAE HSL2*-independent categories. For example there are *HAE HSL2*-independent defense response, lignin biosynthesis, and suberin biosynthesis genes. There are even some *HAE HSL2*-independent hydrolases, particularly XTHs, which may explain the observation that *hae hsl2* and *ida* mutants initially undergo a decrease in the force required to remove the floral organs before increasing again [6].

## Conclusions

Abscission is a tightly regulated process resulting in the breakdown of the middle lamella at the AZ and shedding of the abscising organ. This process is regulated by the two RLKs HAE and HSL2 which operate through a MAP kinase cascade. However the targets of this signaling pathway were previously unknown. Our work reveals the HAE HSL2 signaling regulates the expression of hydrolytic and cell wall modifying enzymes necessary for the breakdown of the pectin rich middle lamella and the expression of defense response genes during abscission. Disruption of HAE HSL2 signaling prevents normal expression of these genes, resulting in the loss of abscission phenotype observed in *hae hsl2* mutants. These genes form three distinct expression clusters with most genes having peak expression in late stage 15, right before organ separation. Testing of differentially expressed glycosyl hydrolases in *ida* mutants shows that many of the same genes are co-regulated by IDA and HAE HSL2 and support the role of IDA in the HAE and HSL2 signaling pathway. Comparison to stamen AZ microarrays shows there are *HAE HSL2*-independent processes that includes the ethylene, abscissic acid, and jasmonic acid hormonal signaling pathways. Responses of these pathways, such as senescence and callose deposition increase prior to abscission, while other processes like cellulose synthase decrease. This demonstrates the complexity of abscission and how multiple pathways integrate to lead to organ loss.

## Methods

### Plant materials and growth conditions

*Arabidopsis thaliana* Columbia-0 wild type, *hae-3 hsl2-3*, and *ida-2* plants were grown in growth chambers at 22C under 16 h light 8 h dark. At 7 weeks of age, stage 15 flowers were collected and the flower receptacles were immediately dissected and frozen in liquid nitrogen. Receptacles were dissected by cutting off the stems from the base of the flower and slightly above the base of the stamen with a surgical knife. 20 receptacles were pooled per sample, each receptacle from a separate plant. All samples were collected between 4 and 6 pm to minimize circadian effects. For qPCR this was repeated with stage 12, 13, and 14 flower receptacles collected in addition to stage 15. Two sets of wild type samples were collected for qPCR, one grown alongside *hae hsl2* the other grown later alongside *ida*.

### RNA-Seq library construction

RNA was isolated using TRIZOL reagent (Invitrogen). DNA was removed using DNase TURBO (Ambion) and then cleaned up using RNAeasy Mini Kit (Qiagen). RNA-Seq libraries were prepared using the TruSeq RNA sample preparation kit (Illumina) following the manufacturer’s protocol. All six samples were pooled together and run on three lanes, each on a separate flow cell of an Illumina HiSeq 2000.

### Read mapping and differential expression

Reads were quality trimmed using FASTX FASTQ Quality Trimmer version 0.0.13 [35] with a minimum quality score of 13 and a minimum length of 32. Because adaptor sequence was present at the three prime end of some reads, these were further trimmed using CUTADAPT version 0.9.5 [36] with a minimum overlap of 2 and a minimum length of 32. Reads were then aligned back to the TAIR10 version of the Arabidopsis genome [37] using TOPHAT version 1.3.1 [38] supplied with the TAIR10 GFF at default settings. Sam and bed files were generated using SAMtools version 0.1.18 [39] and BEDTools version v2.12.0 [40]. Read counts for each gene was quantified using HTseq-count with the settings stranded=no, mode=union, and type=gene [41].

Differential Expression was determined using the DESeq version 1.5.6 [21]. This was done using the sequence of commands: newCountDataSet, estimateSizeFactors, estimateDispersions, and nbinomTest. For the estimateDispersions function the settings used are method = “per-condition”, sharingMode = “maximum”, fitType = “parametric”.

### Gene ontology

Gene Ontologies were analyzed for term enrichment using the agriGO Single Enrichment Analysis tool [42] with TAIR10 GO annotations. The full GO contains thousands of specific terms while the GO Slim is a reduced set of broader higher level terms that is easier to represent graphically. Both the full GO and GO Slim were used. The hypergeometric test was used with Benjamini-Hochberg FDR correction and a p < 0.05. Lower expressed and higher expressed genes were analyzed separately.

### qPCR

Primers for qPCR (Additional file 10) were designed using Primer3 software [43] to have a Tm of 59-61 C, with a length of 22-26 nts and a product length of 60-150 bps [44]. Reference genes were chosen from Czechowski et al. [45] for stability across tissue type and developmental stage. These were then checked against RNA-Seq results for stability in the *hae hsl2* background. RNA was isolated using TRIZOL reagent (Invitrogen) and DNA was removed using DNase TURBO (Ambion). Samples were then cleaned up using RNAeasy Mini Kit (Qiagen). 1000 ng of RNA was used to make cDNA with the SuperScript III First Strand Synthesis Kit (Invitrogen). 200 nm of each primer were added to each well and dried overnight. For qPCR 2.5 uL of Platinum SYBR Green qPCR Supermix-UDG (Invitrogen) and 1 uL of cDNA (1 ng/uL) for a total of 5 uL was used in each well. To reduce pipetting errors master mixes of the cDNA and 2x reaction mix were made prior to dispensing. For each sample 3 biological reps were used and repeated 3 times for technical replication. Real-time PCR was done on a CFX384 Touch Real-Time PCR Detection System (BioRad) at 50C for 2 minutes, 95C for 2 mins, and 45 cycles of 95C for 15 s, 55C for 30s, and 72C for 30s followed by a melting curve analysis. qPCR was analyzed on CFX Manager Software (BioRad) using the ΔΔCt method [46]. Statistical significance was determined using Student’s t-test.

### Reanalysis of microarray data

Microarray data from Cai and Lashbrook [14] was downloaded from Array Express [47] and reanalyzed using ROBIN [48] using the probe logarithmic error intensity estimate (PLIER) algorithm [49] and the linear models from the limma package [50]. Stage 12 was used as a baseline of comparison to later stages resulting in the following stage categories: stage 12-stage 13, stage 12-early stage 15, stage 12-mid stage 15, and stage 12-late stage 15. Differentially expressed genes were considered significant with a FDR adjusted p < 0.05.

### Cluster analysis

Microarray signal intensities extracted for the list of differentially expressed genes in *hae hsl2* were normalized to a range of -1 to 1 and centered on a mean of 0 using Cluster 3.0 [51]. These were imported into R and the number of k-Means clusters was estimated to be 3 using the gap statistic gap [26]. Final clustering was done by k-Means clustering in Cluster 3.0 using Euclidean distance as the Similarity Metric, a k = 3, and 1000 runs.

### Comparison of microarrays and RNA-Seq

Because RNA-Seq detects a broader range of transcripts than microarrays, which are limited to the probe set, any genes not found on the array were first filtered from the RNA-Seq data. Log2 fold changes of differentially expressed genes in both RNA-Seq and mircoarray data was then compared using VennMapper software [52] looking for overlap in genes with a 2 fold or greater difference in expression. Genes that were expressed at lower and higher quantities in hae hsl2 relative to wild type were treated as separate categories and compared in a pairwise fashion to each stage.

### Data availability

Raw reads, bed alignment files, and raw gene counts, are available under the GEO accession number GSE35288 at the NCBI Gene Expression Omnibus [53].

## Abbreviations

RLK: Receptor-Like Kinase; HAE: HAESA; HSL2: HAESA-LIKE 2; AZ: Abscission Zone; IDA: INFLORESCENCE-DEFICIENT IN ABSCISSION; MKK4: MAP KINASE KINASE 4; MKK5: MAP KINASE KINASE 5; MPK3: MAP KINASE 3; MPK6: MAP KINASE 6; PG: Polygalacturonase; PGAZAT/ADPG2: POLYGALACTURONASE ABSCISSION ZONE A. THALIANA/ARABIDOPSIS DEHISCENCE ZONE POLYGALACTURONASE 2; QRT2: Quartet 2; FDR: False Discovery Rate; GO: Gene Ontology; FAR4: FATTY ACYL-COENZYME A REDUCTASE 4; FAR5: FATTY ACYL-COENZYME A REDUCTASE 5; XTH: Xylogulcan endo-Transglycosylase/Hydrolases; SAMI: S-ADENOSYLMETHIONINE SYNTHETASE 1; ACS8: 1-AMINO-CYCLOPROPANE-1-CARBOXYLATE SYNTHASE 8; ACO1: ACC OXIDASE 1; ACO2: ACC OXIDASE 2; ETR2: ETHYLENE RESPONSE 2; ABI2: ABA INSENSITIVE 2; ABI4: ABA INSENSITIVE 4; ABI5: ABA INSENSITIVE 5; AOC3: ALLENE OXIDE CYCLASE 3; AOC4: ALLENE OXIDE CYCLASE 4; LOX2: LYPOXYGENASE 2; LOX4: LYPOXYGENASE 4; CESA5: CELLULOSE SYNTHASE 5; CESA7: CELLULOSE SYNTHASE CATALYTIC SUBUNIT 7; EIN2: ETHYLENE INSENSITIVE 2; ABA2: ABA DEFFICIENT 2; EIN3: ETHYLENE INSENSITIVE 3.

## Competing interests

The authors declare they have no competing interests.

## Authors’ contributions

C.E.N. contributed to experimental design, data collection, data analysis and writing of the paper. O.R.P. contributed to experimental design, data collection and commented on the paper. J.C.W. contributed to experimental design, data analysis and writing of the paper. All authors read and approved the final manuscript.

## Supplementary Material

Additional file 1 Stage 12-17 flowers and dissected receptacles for stages 12-15. Click here for file

Additional file 2 **DESeq results. (A)** All genes **(B)** Differentially expressed genes in RNA-Seq with a FDR adjusted p < 0.05 and fold change > 2 fold. Click here for file

Additional file 3 Full GO term-enrichment for RNA-Seq differentially expressed genes. Click here for file

Additional file 4 Cluster assignments of differentially expressed genes and microarray mean-normalized expression values used in clustering. Click here for file

Additional file 5 Gap Statistic for different numbers of k-Means clusters. Click here for file

Additional file 6 **Full GO term-enrichment for individual clusters. (A)** Cluster 1 **(B)** Cluster 2 **(C)** Cluster 3. Click here for file

Additional file 7 **qPCR across developmental stages 12 (white), 13 (light grey), 14 (dark grey), 15 (black). (A)** Wild Type 1 **(B)***hae hsl2 ***(C)** Wild Type 2 **(D)***ida*. (*) lower than stage 15 (**) higher than stage 15. Click here for file

Additional file 8 **(A)** Number of genes overlapping between RNA-Seq and Stamen AZ microarrays. **(B)** Genes overlapping between RNA-Seq and Stamen AZ microarrays. **(C)** Z-scores of sample overlap between RNA-Seq and Stamen AZ microarrays. Click here for file

Additional file 9 **Full GO term-enrichment for stage 12-late stage 15 differentially expressed genes that do not overlap with RNA-seq data. (A)** Genes showing higher expression from stage 12-late stage 15 **(B)** Genes showing lower expression from stage 12-late stage 15. Click here for file

Additional file 10 Primer sequences used in qPCR. Click here for file
