# Supplementary figures and images for: Transcriptional profiling of the Arabidopsis abscission mutant hae hsl2 by RNA-Seq
Source: BMC Genomics. 2013 Jan 17;14:37. doi: 10.1186/1471-2164-14-37 (PMC3566969; doi:10.1186/1471-2164-14-37)

**A**

**Flower Stage**

**12**

**13**

**14**

**15**

**16**

**17**

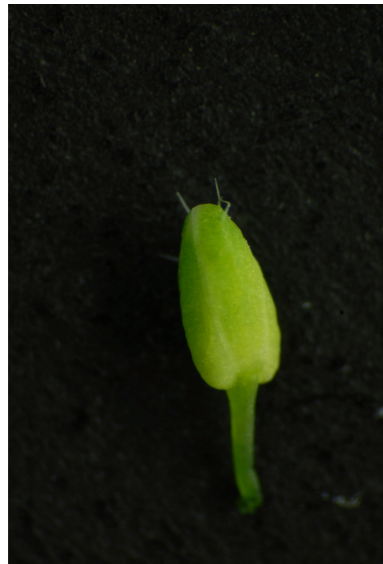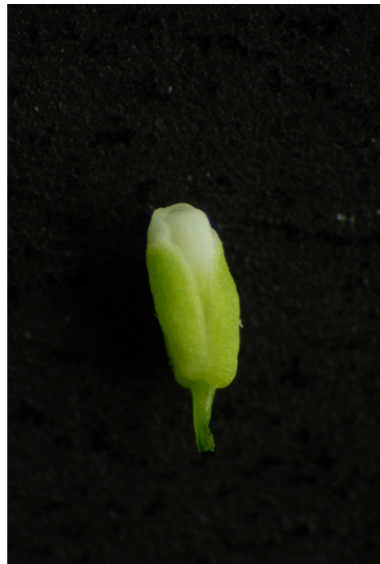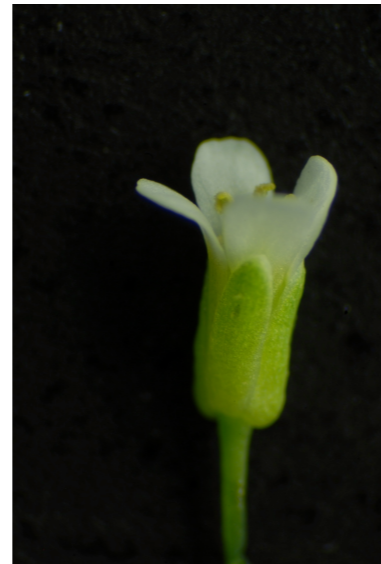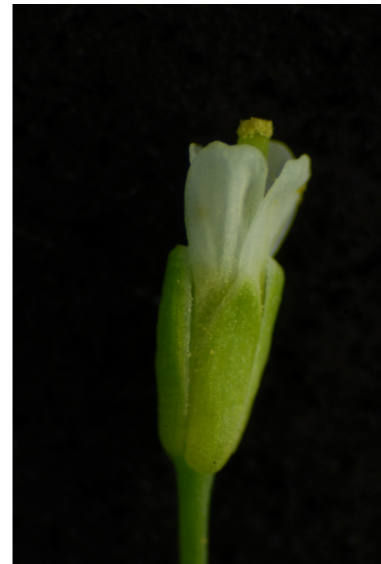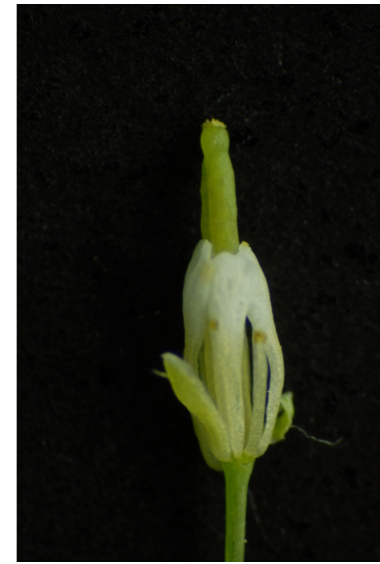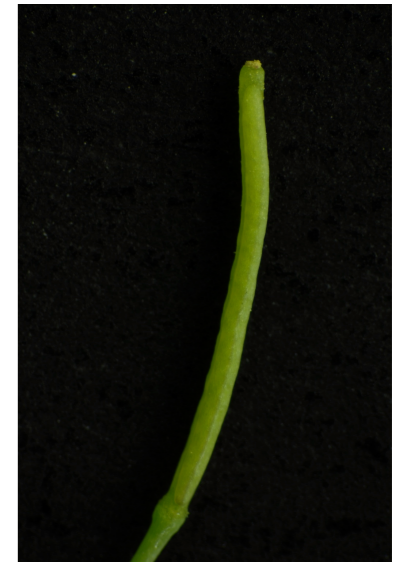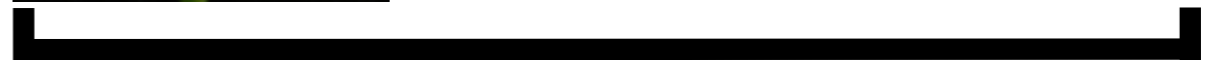

**B**

**Abscission**

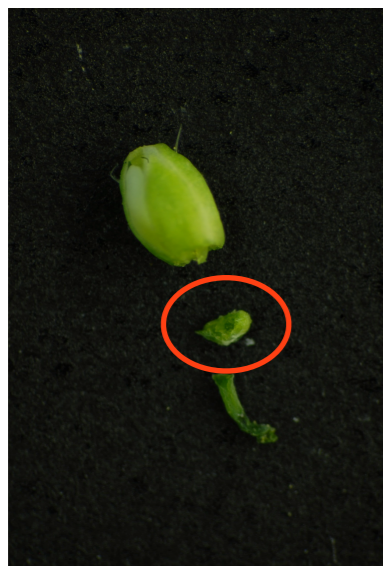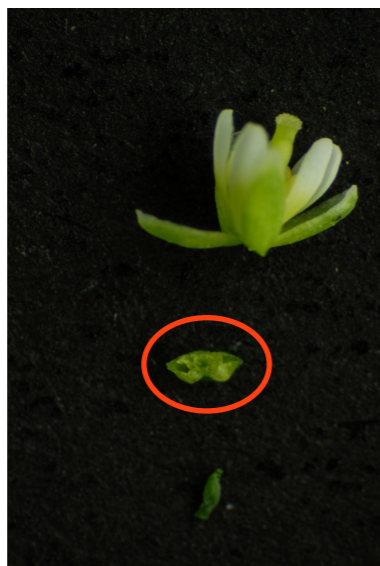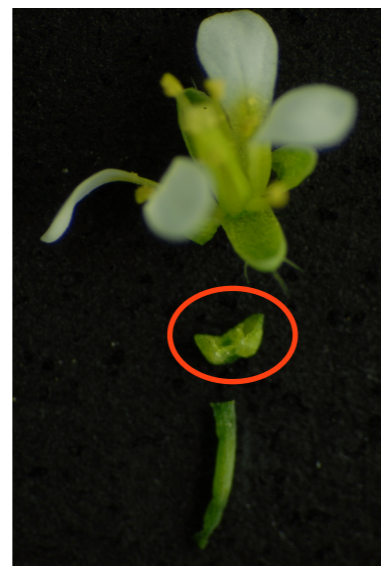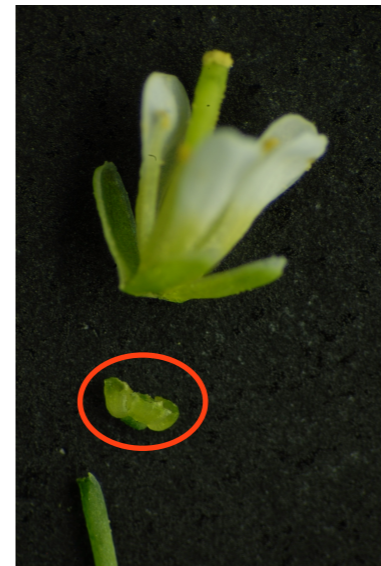

**Dissected flower receptacles**

Supplement: Additional file 1 — Stage 12-17 flowers and dissected receptacles for stages 12-15. [file 1471-2164-14-37-S1.pdf]

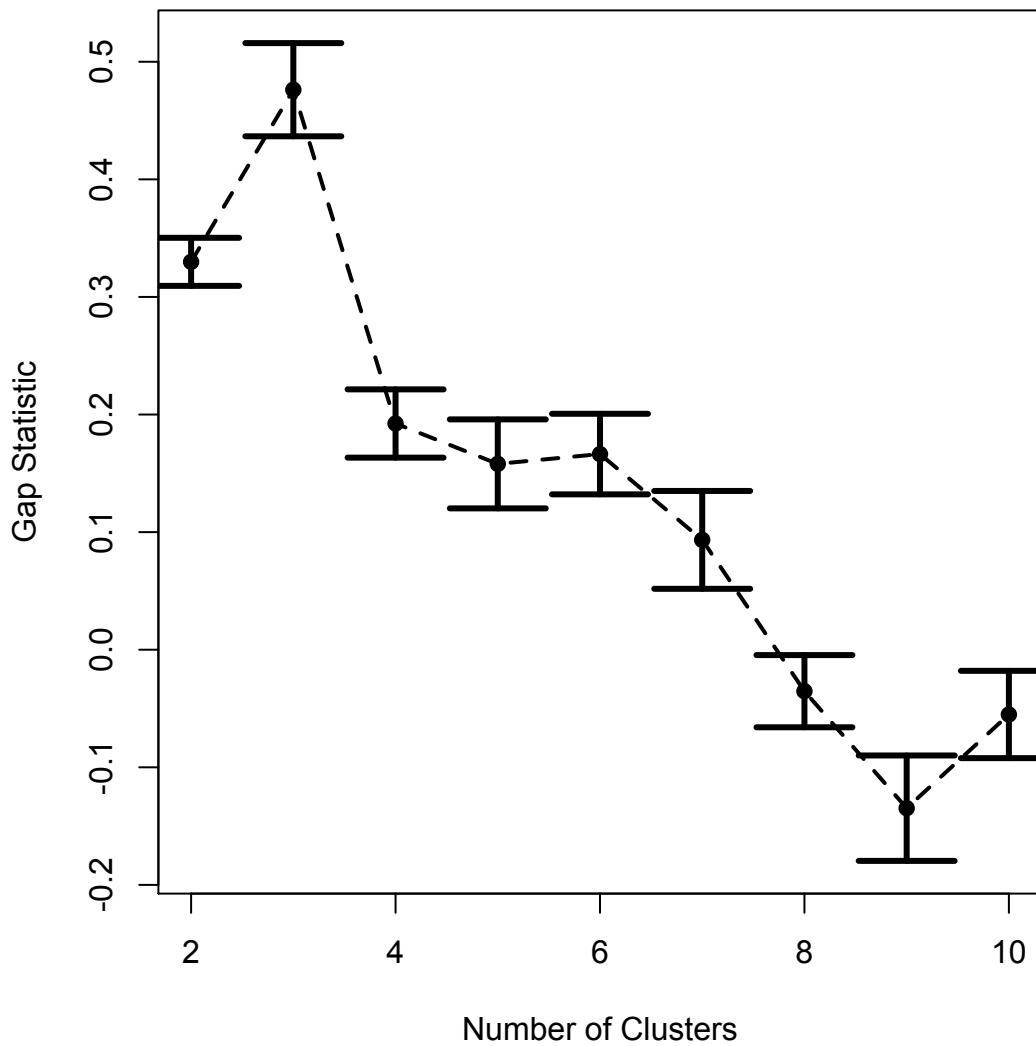

Supplement: Additional file 5 — Gap Statistic for different numbers of k-Means clusters. [file 1471-2164-14-37-S5.pdf]

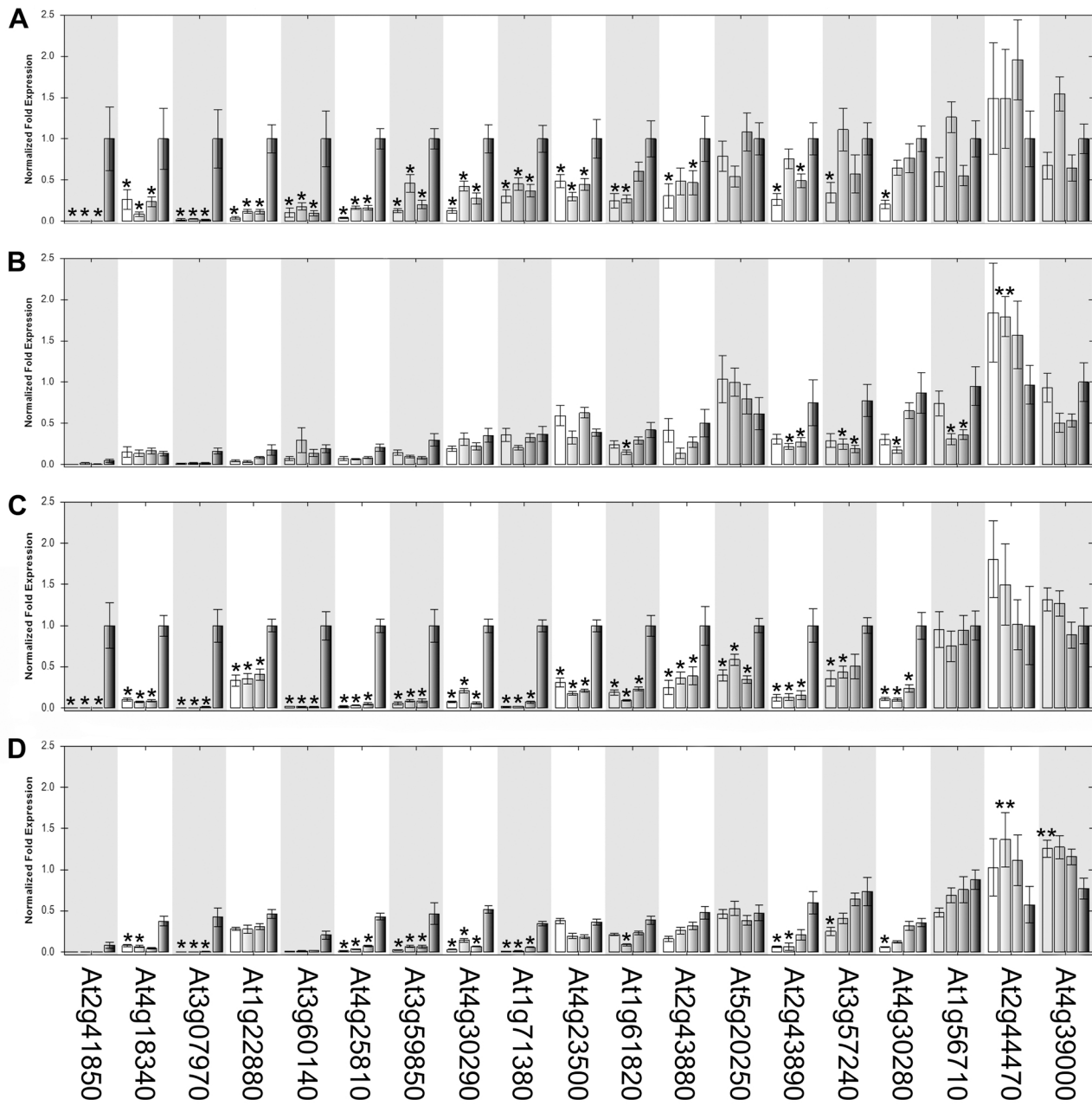

Supplement: Additional file 7 — qPCR across developmental stages 12 (white), 13 (light grey), 14 (dark grey), 15 (black). (A) Wild Type 1 (B)hae hsl2 (C) Wild Type 2 (D)ida. (*) lower than stage 15 (**) higher than stage 15. [file 1471-2164-14-37-S7.pdf]
